# Thalamic volume in very preterm infants: associations with severe brain injury and neurodevelopmental outcome at two years

**DOI:** 10.3389/fneur.2024.1427273

**Published:** 2024-08-14

**Authors:** Emiliano Trimarco, Bahram Jafrasteh, Natalia Jiménez-Luque, Yolanda Marín Almagro, Macarena Román Ruiz, Manuel Lubián Gutiérrez, Estefanía Ruiz González, Antonio Segado Arenas, Simón Pedro Lubián-López, Isabel Benavente-Fernández

**Affiliations:** ^1^Biomedical Research and Innovation Institute of Cádiz (INiBICA) Research Unit, Puerta del Mar University Hospital, Cádiz, Spain; ^2^Division of Neonatology, Department of Paediatrics, Puerta del Mar University Hospital, Cádiz, Spain; ^3^Area of Paediatrics, Department of Child and Mother Health and Radiology, Medical School, University of Cádiz, Cádiz, Spain

**Keywords:** thalamus, preterm infants, neurodevelopment, brain injury, MRI

## Abstract

**Introduction:**

Several studies demonstrate the relationship between preterm birth and a reduced thalamus volume at term-equivalent age. In contrast, this study aims to investigate the link between the thalamic growth trajectory during the early postnatal period and neurodevelopment at two years of age.

**Methods:**

Thalamic volume was extracted from 84 early MRI scans at postmenstrual age of 32.33 (± 2.63) weeks and 93 term-equivalent age MRI scans at postmenstrual age of 42.05 (± 3.33) weeks of 116 very preterm infants (56% male) with gestational age at birth of 29.32 (± 2.28) weeks and a birth weight of 1158.92 (± 348.59) grams. Cognitive, motor, and language outcomes at two years of age were assessed with Bayley Scales of Infant and Toddler Development Third Edition. Bivariate analysis was used to describe the clinical variables according to neurodevelopmental outcomes and multilevel linear regression models were used to examine the impact of these variables on thalamic volume and its relationship with neurodevelopmental outcomes.

**Results:**

The results suggest an association between severe brain injury and thalamic growth trajectory (β coef = −0.611; *p* < 0.001). Moreover, thalamic growth trajectory during early postnatal life was associated with the three subscale scores of the neurodevelopmental assessment (cognitive: β coef = 6.297; *p* = 0.004; motor: β coef = 7.283; *p* = 0.001; language: β coeficient = 9.053; *p* = 0.002).

**Discussion:**

These findings highlight (i) the impact of severe brain injury on thalamic growth trajectory during early extrauterine life after preterm birth and (ii) the relationship of thalamic growth trajectory with cognitive, motor, and language outcomes.

## 1 Introduction

Very preterm infants (VPI), those born at or before 32 weeks of gestational age (GA), have a higher risk of mortality and adverse neurodevelopmental outcome (1–3). Despite improvements in survival rates over the past few decades including for extremely preterm infants (4–6), preterm birth continues to impact negatively on long-term neurodevelopmental outcomes (7–10). Crucial brain development events that take place in the third trimester of pregnancy may be disrupted and modified by preterm birth with subsequent risk of neurological injury (11–13). Preterm delivery and its consequences can impact specific aspects of cerebral development, such as the thalamocortical system (14–17), affecting thalamic volume and white matter maturation even in the absence of brain injury (18). Thus, thalamic volume in premature newborns has been associated with the type of nutrition (19), the presence of white matter injury (20–23), exposure to painful procedures (24), parenting (25). Recent research by our group demonstrated the potential to cluster magnetic resonance images (MRI) deemed pathological according to Kidokoro's score (26) using only morphological thalamus variables extracted from term-equivalent age (TEA) T1-weighted scans (27). Abnormalities in thalamocortical connectivity and reduced thalamic volume have been linked to adverse neurodevelopmental outcomes (28–30) and lower cognitive performance in adulthood (31, 32) in very preterm population. Furthermore, abnormalities in thalamocortical connectivity explored with functional MRI have been associated with Autism Spectrum Disorder (33, 34). While numerous studies have explored various aspects of early (35, 36) and TEA (14, 28, 29) brain scans, there is a notable lack of research investigating the combination of these scans using volume growth trajectory analysis. Our study aims to address this gap by providing insights into the neurodevelopmental processes from early to TEA, thereby contributing to a deeper understanding of brain development during this critical period. Using a trajectory instead of just a point provides a dynamic and comprehensive measure of brain development by capturing changes in thalamic volume over time, as opposed to a single static measurement. This approach allows for the identification of growth patterns, offering valuable insights into developmental processes and potential abnormalities that might be missed with a single volume measure. Therefore, our hypothesis seeks to investigate the relationship between clinical complications, such as brain injury, and the thalamic volume growth trajectory measured at two distinct time points. Additionally, we aim to explore the potential of utilizing the thalamic volume growth trajectory as a predictive marker for neurodevelopmental outcomes at two years of age in a population of VPI. This investigation involves a comprehensive analysis of thalamic volume changes over time, considering various clinical factors that may influence neurodevelopmental outcomes.

## 2 Material and methods

### 2.1 Participants

**Figure 1:**
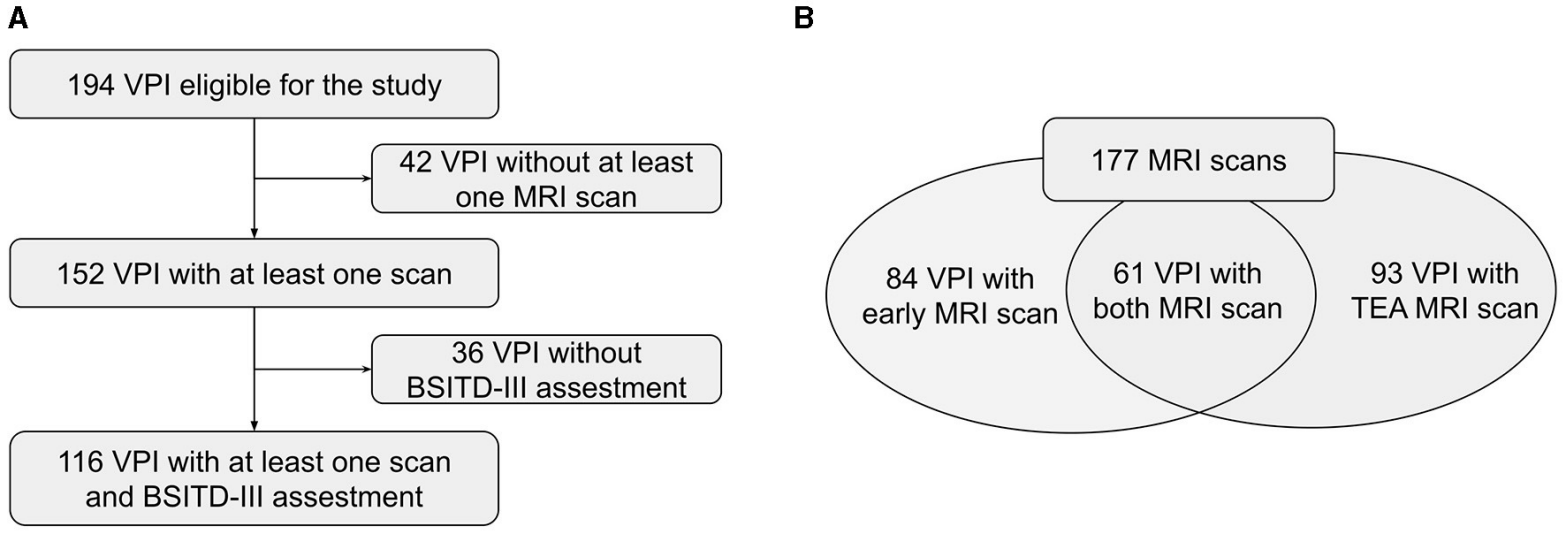
**(A, B)** Flow diagram of the study population. VPI, Very Preterm Infants; MRI, Magnetic Resonance Image; BSITD-III, Bayley Scales of Infant and Toddler Development, Third Edition; TEA, Term-Equivalent Age.

This longitudinal study included VPI, with GA at birth ≤ 32 weeks, admitted to the Hospital Puerta del Mar, Cádiz, Spain, from January 2018 to September 2021. The exclusion criteria were defined as the presence of congenital or chromosomal anomalies, metabolic disorders, and central nervous system infections. This study was approved by the Research and Ethics Committee, and all parents or guardians of the participants provided informed consent. The data used in this study were collected and managed using Research Electronic Data Capture (REDCap) software (37, 38). The initial cohort included 194 eligible infants, of which 152 had at least one MRI. We excluded 36 patients who did not complete neurodevelopmental assessment at 2 years, with a final sample size of 116 preterm infants with at least one MRI and 2-year neurodevelopmental assessment (Figure 1A). VPI not included in the final study sample (n= 78) did not differ significantly in GA, birth weight, and sex from those included, as reported in Table 1. We included 177 brain MRIs: 84 (47.5%) were early scans at a mean PMA of 32.33 (± 2.63) weeks, and 93 (52.5%) TEA scans were performed at a mean PMA of 42.05 (± 3.33) weeks. Remarkably, 61 of 116 infants (52.6%) had both early and TEA scans (Figure 1B). Our final study sample included 116 VPI (56% male) with a mean GA at birth of 29.32 (± 2.28) weeks and a mean birth weight of 1158.92 (± 348.59) grams.

**Table 1:** Comparison between very preterm infants included in the study and ones excluded.

	**VPI included (*n* = 116)**	**VPI excluded (*n* = 78)**	***p***
GA (weeks)	29.3 ± 2.3	28.2 ± 2.8	0.621
Sex (Male)	65 (56.0 %)	39 (50.0 %)	0.409
Weight (grams)	1,159 ± 349	1,036 ± 383	0.594

VPI, very preterm infants; GA, gestational age. Continuous variables are presented as mean ± standard deviation; Binary variables are shown as an absolute and relative frequency (percentage).

### 2.2 MRI acquisition and analysis

**Figure 2:**
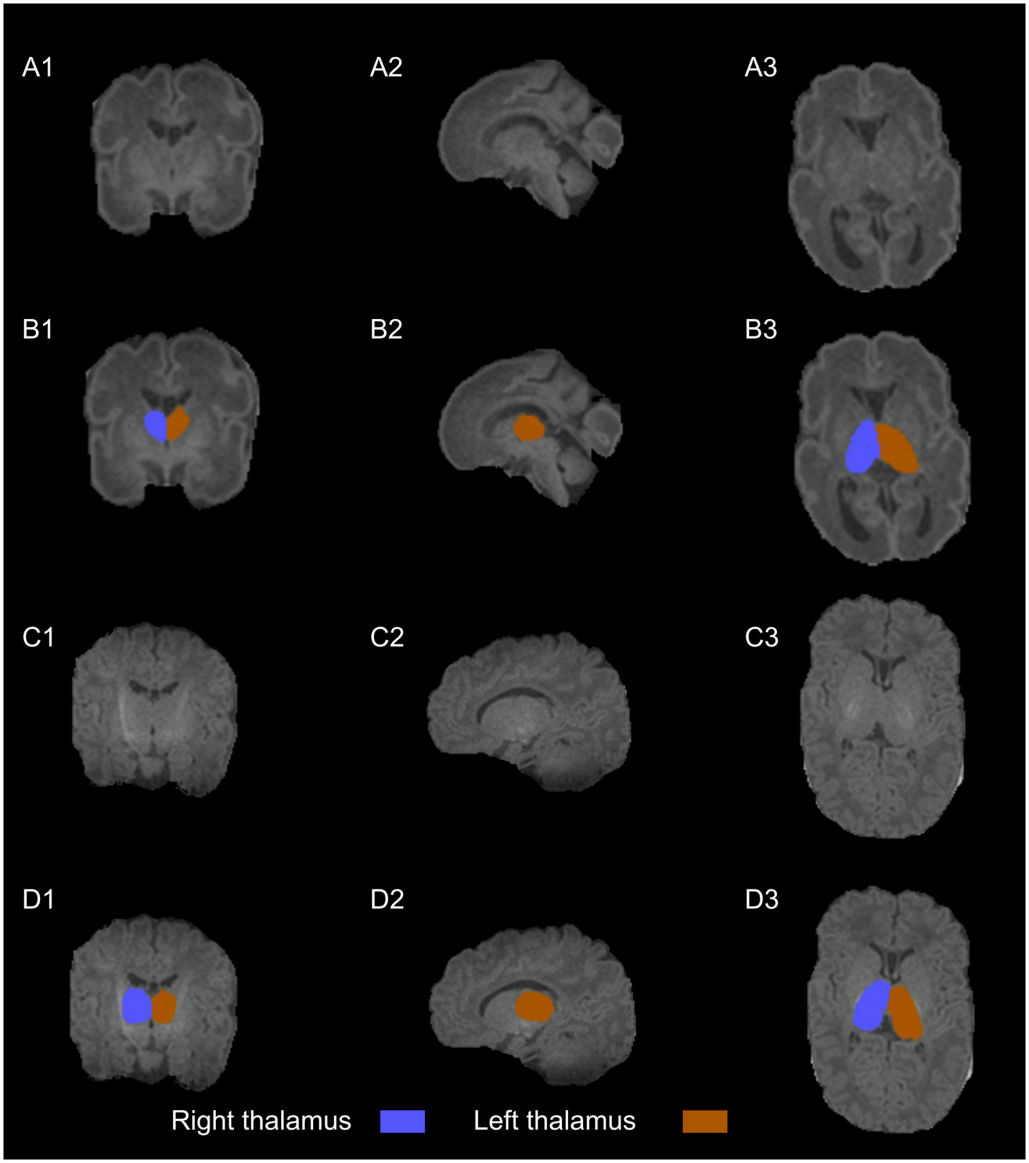
T1w images **(A–C)** with thalamus segmentation **(B–D)** into coronal (1), sagittal (2), and axial planes (3). The scans are from a healthy very preterm infant of 29.14 weeks of gestational age. The early scan **(A, B)** shows a right thalamus volume (blue) of 1.75 cm^3^ and a left thalamus volume (orange) of 1.80 cm^3^ at 27.57 weeks of postmenstrual age while the TEA scan **(C, D)** shows a right thalamus volume (blue) of 4.45 cm^3^ and a left thalamus volume (orange) of 4.47 cm^3^ at 43.14 weeks of postmenstrual age.

MRI scans were performed at 2 chronological points: in the first weeks of life and at the TEA. MRI scans were performed using a 1.5 T scanner Magneton Symphony (Siemens Health Care, Erlangen, Germany) located in the radiology unit. T1w images were obtained using a three-dimensional spoiled gradient [repetition time 1,660 (RT)/echo time 5.16(ET)]. The acquisition parameters were as follows: spacing in x, y and z direction: 0.54, 0.54, 1.09; echo time=3.67 ms; flip angle=15° and repetition time=1910.0 ms. The obtained T1w MRI has been converted to equal spacing of 0.9 for study purpose. MRI preprocessing included automatic brain extraction followed by manual correction, N4 bias field correction, and image enhancement and it was performed using MELAGE (39). The results were verified by experts and manually corrected if needed. The thalamus segmentation was performed using an approach from our previous work (27) and was further refined by experts in the group. Specifically, we employed a multi-atlas approach using the Melbourne Children's Regional Infant Brain (M-CRIB 2.0) atlas (40). We first registered the MRI images to the neonatal atlas and then transferred the atlas labels to the MRI images. Finally, we used a local atlas weighting to assign a label to the thalamus, similar to the neonatal pipeline proposed by (41, 42). Figure 2 illustrates an example of early and TEA MRI images with overlaid segmentation. From left to right, we can see coronal, sagittal, and axial views, respectively. After the left and right thalamic volume segmentions, we tested the correlation between them. The Pearson correlation coefficient was very high (*r* = 0.98), indicating a very strong linear relationship and suggesting that the two volumes are almost identical in our study population. Therefore, the sum of left and right volumes was used in the statistical analysis as a more robust measure of overall thalamic volume.

### 2.3 Clinical variables

Perinatal and postnatal variables were prospectively collected, including sex, weight at birth, GA, and postmenstrual age (PMA) at the time of the scan. VPI born with a weight below the 10th percentile for the gestational age are defined as small for gestational age (SGA) (43). The comorbidities were grouped in a scale based on other composite medical risk scales (44, 45), including: significant patent ductus arteriosus (PDA) (patent requiring surgical or pharmacological closure), severe retinopathy of prematurity (ROP) (stage 3 or higher), moderate to severe bronchopulmonary dysplasia (BPD) (oxygen requirements at 36 weeks PMA), late-onset sepsis (LOS) (systemic signs of infection and isolation of a bacterial pathogen in blood culture after 5 days of life) and necrotising enterocolitis (NEC) (Bell stage II or higher). The presence of at least one comorbidity was scored with 1, and absence was scored with 0. Severe brain injury was considered in the presence of grade 3 germinal matrix-intraventricular hemorrhage (GM-IVH 3) and/or parenchymal hemorrhagic infarction (PHI), and/or moderate to severe white matter injury (WMI) (46). Maternal years of education were considered an approximation to socioeconomic status (47, 48).

### 2.4 Neurodevelopmental outcomes

At two years of corrected age, assessments were performed using the Bayley Scales of Infant and Toddler Development, Third Edition (BSITD-III) (49). The cognitive, motor, and language scale scores are standardized to a mean of 100 and a standard deviation of 15. In our study sample, the mean scores obtained were 100.26 (± 14.74) for cognitive, 98.80 (± 14.20) for motor and 96.48 (± 17.25) for language scales. The three scores were considered both quantitative measurements and qualitative ones, considering a favorable outcome as a score ≥ 85, while an adverse outcome was considered for a score < 85. Among the VPI included in the study, 16 (13.8%) showed an adverse cognitive outcome, 19 (16.4%) an adverse motor outcome and 22 (19.0%) an adverse language outcome.

### 2.5 Statistical analysis

The initial phase involves a descriptive analysis of the clinical variables of VPI, comparing those exhibiting favorable outcomes to those with adverse outcomes for each BSITD-III subscale. Variables were described as frequency and percentage if they were categorical variables. If continuous, they were expressed as mean and standard deviation or median and interquartile range after testing for normality. Bivariate analysis was performed using Pearson's chi-squared test or Fisher's exact test for categorical variables and Student t-test or Mann-Whitney U test for continuous variables. The second step concerns a multilevel linear regression model to study the association between clinical variables and thalamic volume, accounting for repeated measurements and time. In the third stage, multilevel linear regression models were used to investigate the relationship between thalamic volume and clinical variables with each BSITD-III subscale score, accounting for repeated measurements and time. The included variables were selected based on the theoretical background and with a backward stepwise approach to exclude the non-significant variables if not considered variables for which an adjustment was needed. All models are adjusted for GA, PMA at the scan and total brain volume. Statistical analysis was conducted using Stata 17.0 (Stata Statistical Software: Release 17, College Station, TX: StataCorp LP). The results were considered statistically significant at *p* ≥ 0.05.

## 3 Results

### 3.1 Clinical variables and 2-year neurodevelopmental outcome

Adverse cognitive outcome was related to sex, with a higher proportion of males having adverse cognitive outcome compared to good outcome (14 (87.5%) vs. 51 (51.0%); *p*=0.006), significant PDA (5 (31.2%) vs. 9 (9.0%); *p*=0.025), moderate to severe BPD (7 (43.75%) vs. 15 (15.0%); *p*=0.006), grade 3 GM-IVH and/or PHI (6 (37.5%) vs. 6 (6.0%); *p*=0.001) and severe brain injury (7 (43.7%) vs. 8 (8.0%); *p*=0.001). Regarding the motor score, adverse outcome was related to severe ROP (4 (21.0%) vs. 3 (3.1%); *p*=0.013), moderate to severe BPD (9 (47.4%) vs. 13 (13.4%); *p*=0.001), GM-IVH 3 and/or PHI (5 (26.3%) vs. 7 (7.2%); *p*=0.012), moderate to severe WMI (3 (18.7%) vs. 1 (1.0%); *p*=0.014) and severe brain injury (7 (36.8%) vs. 8 (8.2%); *p*=0.001). We also found fewer years of maternal education were associated with adverse motor outcome (13.3 ± 4.4 vs 14.9 ± 3.8; *p*=0.050). Finally, adverse language outcome was associated with being male (18 (81.8%) vs. 47 (50.0%); *p*=0.008), severe ROP (4 (18.2%) vs 3 (3.2%); *p*=0.024), moderate to severe BPD (10 (45.4%) vs 12 (12.8%); *p*=0.001), GM-IVH 3 and/or PHI (5 (22.7%) vs. 7 (7.4%); *p*=0.034), moderate to severe WMI (3 (13.6%) vs. 1 (1.0%); *p*=0.021) and severe brain injury (7 (31.8%) vs. 8 (8.5%); *p*=0.003). In addition, fewer years of maternal education (13.2 ± 3.6 vs. 14.9 ± 3.9; *p*=0.028) were also associated with adverse language outcome. A detailed description of the clinical variables related to the 2-year neurodevelopmental outcome is present in Table 2.

**Table 2:** Clinical variables of VPI with and without adverse outcome.

	**Cognitive outcome**			**Motor outcome**			**Language outcome**			
	**Adverse (*n* = 16)**	**Favorable (*n* = 100)**	***p***	**Adverse (*n* = 19)**	**Favorable (*n* = 97)**	***p***	**Adverse (*n* = 22)**	**Favorable (*n* = 94)**	***p***	**Total (n = 116)**
Sex (male)	14 (87.5%)	51 (51.0%)	0.006^*^	14 (73.7%)	51 (52.6%)	0.129	18 (81.8%)	47 (50%)	0.008^*^	65 (56.0 %)
Weight (grams)	1,075 ± 409	1,172 ± 338	0.150	1,142 ± 336	1,162 ± 353	0.407	1,051 ± 396	1,184 ± 334	0.053	1,159 ± 349
SGA	3 (18.7%)	15 (15.0%)	0.713	3 (15.8%)	15 (15.5%)	1.000	6 (27.3%)	12 (12.8%)	0.107	18 (15.5%)
GA (weeks)	28.3 ± 2.7	29.5 ± 2.2	0.066	29.0 ± 2.4	29.4 ± 2.3	0.395	28.7 ± 2.6	29.4 ± 2.2	0.241	29.3 ± 2.3
Significant PDA	5 (31.2%)	9 (9.0%)	0.025^*^	5 (26.3%)	9 (9.3%)	0.053	5 (22.7%)	9 (9.6%)	0.138	14 (12.1%)
ROP	3 (18.7%)	4 (4.0%)	0.054	4 (21.0%)	3 (3.1%)	0.013^*^	4 (18.2%)	3 (3.2%)	0.024^*^	7 (6.0%)
BPD	7 (43.75%)	15 (15.0%)	0.006^*^	9 (47.4%)	13 (13.4%)	0.001^*^	10 (45.4%)	12 (12.8%)	0.001^*^	22 (19.0%)
LOS	5 (31.2%)	22 (22.0%)	0.524	6 (31.6%)	21 (21.6%)	0.349	6 (27.3%)	21 (22.3%)	0.622	27 (23.3%)
NEC	0 (0.0%)	1 (1.0%)	1.000	0 (0.0%)	1 (1.0%)	1.000	1 (4.5%)	0 (0.0%)	0.190	1 (0.9%)
Comorbidity index	11 (68.7%)	34 (34.0%)	0.012^*^	12 (63.2%)	33 (34.0%)	0.017^*^	12 (54.5%)	33 (35.1%)	0.092	45 (38.8%)
GM-IVH 3 and/or PHI	6 (37.5%)	6 (6.0%)	0.001^*^	5 (26.3%)	7 (7.2%)	0.012^*^	5 (22.7%)	7 (7.4%)	0.034^*^	12 (10.3%)
WMI	2 (12.5%)	2 (2.0%)	0.091	3 (18.7%)	1 (1.0%)	0.014^*^	3 (13.6%)	1 (1.0%)	0.021^*^	4 (3.4%)
Severe brain injury	7 ( 43.7%)	8 (8.0%)	0.001^*^	7 (36.8%)	8 (8.2%)	0.001^*^	7 (31.8%)	8 (8.5%)	0.003^*^	15 (12.9%)
Maternal education (years)	13.2 ± 4.2	14.8 ± 3.8	0.067	13.3 ± 4.4	14.9 ± 3.8	0.050^*^	13.2 ± 3.6	14.9 ± 3.9	0.028^*^	14.9 ± 3.9

SGA, small for gestational age; GA, gestational age; PDA, patent ductus arteriosus; ROP, retinopathy of prematurity; BPD, bronchopulmonary dysplasia; LOS, late-onset sepsis; NEC, necrotising enterocolitis; GM-IVH 3, germinal matrix-intraventricular hemorrhage of grade 3; PHI, parenchymal hemorrhagic infarction; WMI, white matter injury. Continuous variables are presented as mean ± standard deviation; Binary variables are shown as an absolute and relative frequency (percentage). ^*^p < 0.05.

### 3.2 Clinical variables and thalamic volume

**Figure 3:**
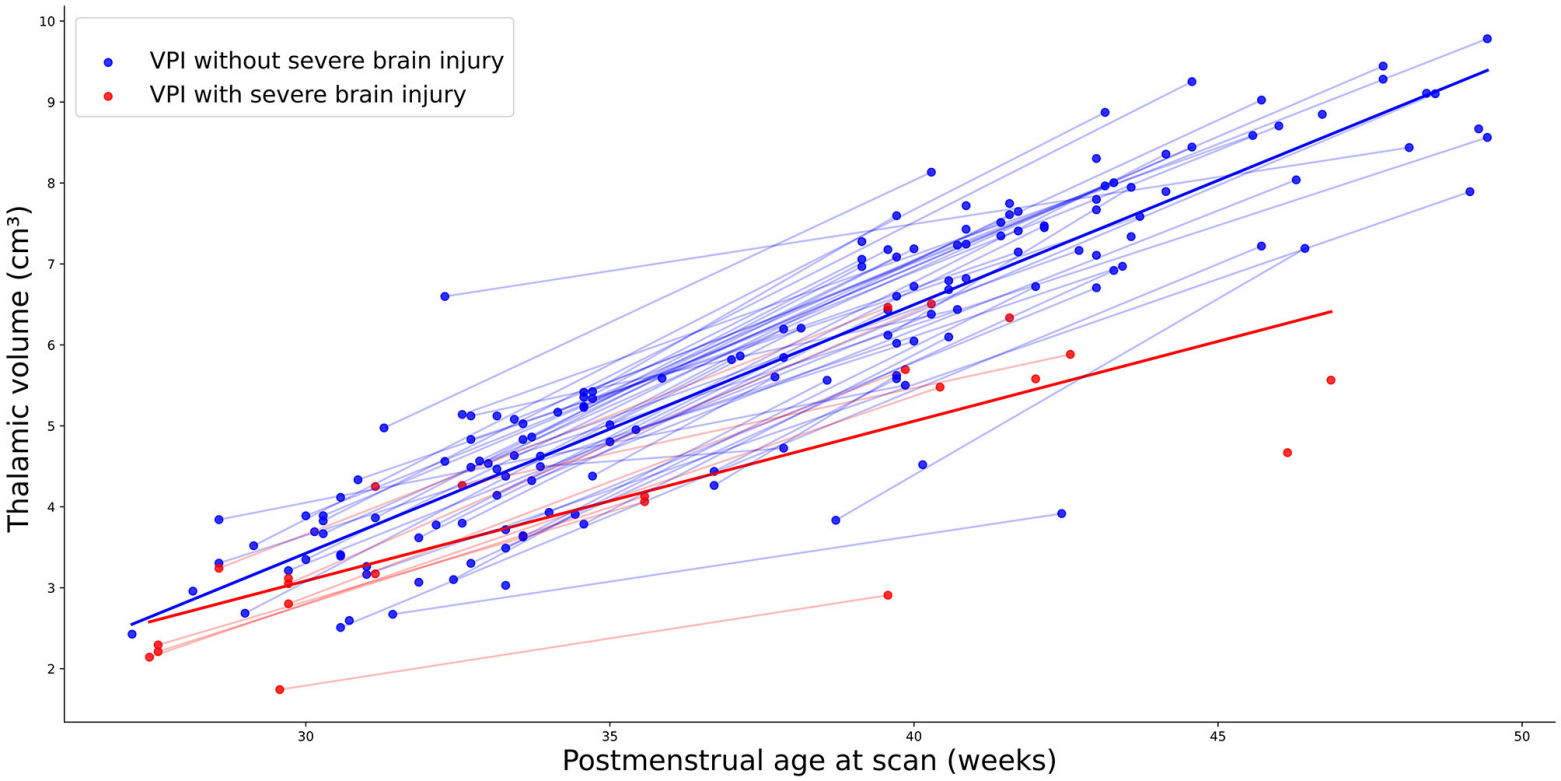
Thalamic growth trajectory in VPI with severe brain injury (red points) and those without severe brain injury (blue points).

When studying the association of thalamic volume with perinatal variable, we found that severe brain injury was significantly related to thalamic volume (β coef=-0.611; *p*= < 0.001). Table 3 summarizes the results of the multilevel regression model and Figure 3 shows the thalamic growth trajectory in VPI with severe brain injury and those without it.

**Table 3:** Results of the multilevel linear regression model based clinical variables in relation to thalamic volume.

**Thalamic volume: Wald chi**^**2**^**=2666.86**, ***p*****-model < 0.0001**		
	**Coef** β	***p***
Const	5.694	< 0.001^*^
GA	0.028	0.212
Sex (Male)	-0.035	0.694
PMA at scan	0.013	0.499
Comorbidity index	-0.056	0.598
Severe brain injury	-0.611	< 0.001^*^
Maternal education (years)	0.008	0.536
Total brain volume	0.015	< 0.001^*^

GA, gestational age; PMA, postmenstrual age. ^*^p < 0.05.

### 3.3 Thalamus volume related to 2-year neurodevelopmental outcome

We studied the relationship of thalamic volume, total brain volume, and perinatal variables with the 2-year neurodevelopmental outcome accounting for repeated measurement and time. Table 4 shows the multilevel model for each BSITD-III subscale. Thalamic growth trajectory during early life was significantly related to cognitive (β coef=6.297; *p*=0.004), motor (β coef = 7.283; *p*=0.001) and language (β coef=9.053; *p*=0.002) outcomes at 2 years of age. Similarly, PMA at scan was related to cognitive (β coef=-1.468; *p*=0.005), motor (β coef=-1.839; *p* < 0.001) and language (β coef=-1.541; *p*=0.004) outcomes. Moreover, years of maternal education were related to cognitive outcome (β coef=0.946; *p*=0.001) and sex (male) was related to language outcome (β coef=-9.091; *p*=0.002).

**Table 4:** Thalamic volume and clinical variables related to 2-year neurodevelopment outcomes.

	**Cognitive**		**Motor**		**Language**	
	**Coef β**	***p***	**Coef β**	***p***	**Coef β**	***p***
Const	102.646	< 0.001^*^	103.768	< 0.001^*^	102.487	< 0.001^*^
GA	0.494	0.376	0.296	0.564	0.535	0.453
Sex (male)	-3.320	0.164	-4.209	0.063	-9.091	0.002^*^
PMA at scan	-1.468	0.005^*^	-1.839	< 0.001^*^	-1.541	0.004^*^
Comorbidity index	0.171	0.950	-4.206	0.099	0.647	0.862
Severe brain injury	-6.098	0.102	-5.254	0.161	-4.787	0.330
Maternal education (years)	0.946	0.001^*^	0.521	0.103	0.743	0.080
Total brain volume	-0.023	0.530	-0.026	0.477	-0.065	0.169
Thalamic volume	6.297	0.004^*^	7.283	0.001^*^	9.053	0.002^*^

GA, gestational age; PMA, postmenstrual age. ^*^p < 0.05.

## 4 Discussion

Our findings align with previous studies that have demonstrated a significant relationship between the presence of severe brain injury and reduced thalamic volume in VPI (14, 50). Srinivasan et al. (14) reported significantly reduced thalamic and lentiform volumes in VPI at TEA compared to term-born controls, with the most marked reductions observed in those with brain injury. Similarly, Kersbergen et al. (50) reported a reduced volume of the thalamus in the TEA scan but not in the early ones of VPI with a specific type of white matter lesion, the cystic periventricular leukomalacia. In addition, some studies indicated a significant relationship between thalamic morphology and neurodevelopment (28, 51). Kline et al. (51) found that thalamic volume, together with temporal lobe curvature and insula curvature, were significant predictors of motor development by analyzing structural brain MRI data at TEA and relating it to BSITD-III motor scores at two years of age in a cohort of 75 VPI. In our study, which includes a larger sample size, we demonstrate a significant relationship between thalamic growth trajectory and various developmental outcomes, including cognitive, motor, and language abilitiess. The study by Loh et al. (28) reported smaller volumes of basal ganglia and thalamic in very preterm compared to term-born infants at TEA. It also establishes a positive relationship between neonatal basal ganglia and thalamic volumes and 7-year neurodevelopmental outcomes, underlining the persistent effect among the lifespan of a reduced thalamic volume at birth. Regarding early scan, Pagnozzi et al. (35) identified cortical gray matter volumes, cortical thickness, and sulcal depth as early predictors of the cognitive and motor outcome at 2 years of age through a prospective cohort of 181 infants with MRI data acquired between 29-35 weeks of PMA. Analogously, Moeskops et al. (36) examined the predictive capacity of automatic quantitative brain MRI descriptors for identifying preterm infants at risk of low cognitive and motor outcomes at 2-3 years of age utilizing MRI scans from 173 VPI acquired between 30 and 40 weeks of PMA. Both early scan studies didn't consider the volume of the thalamus in isolation, but rather, it is grouped with other regions of deep gray matter.

Notably, most studies focusing on morphological MRI analyses often concentrate on scans acquired at TEA, whereas only a few studies consider early scans, and none examine both early scans and TEA scans together. Our work underscores the advantage of studying MRI data from different time points in early extrauterine life and offers insights into thalamic growth trajectory and its impact on neurodevelopment in preterm infants. While some studies adjust their analyses for socioeconomic factors like the mother's educational level, few incorporate potential comorbidities commonly affecting VPI into their analyses. Incorporating such comorbidities into analyses can provide a more comprehensive understanding of the factors influencing outcomes in the preterm population.

This study has several limitations that should be considered: the sample size was relatively small, and future studies with larger cohorts are warranted to validate and generalize the findings. The study focused on thalamic volume, and it would be valuable to consider other brain regions and their interactions in future investigations. Our analyses do not account for potential other confounding factors, such as pain procedures during the early extrauterine life, which has been related to regionally-specific alterations in the thalamus (24) and later adverse neurodevelopment outcome (52).

Moving forward, our research aims to expand in several directions to enhance the depth and scope of our findings. Firstly, we plan to increase the sample size to improve the statistical power and generalizability of our results. Additionally, we intend to extend our investigation beyond infancy and include assessments of neurodevelopmental outcomes during preschool and school-age years. This longitudinal approach will allow us to examine the long-term impact of thalamic growth trajectory in early extrauterine life. Furthermore, we plan to integrate data from other MRI sequences to gain a more comprehensive understanding of brain structure and function. By incorporating information from different imaging modalities, such as diffusion tensor imaging, we can explore additional aspects of brain maturation and connectivity that may contribute to neurodevelopmental outcomes.

## 5 Conclusion

The present work shows the impact of severe brain injury on thalamic growth trajectory during early extrauterine life after preterm birth and the relationship with 2-year cognitive, motor, and language outcomes. Our study highlights how a reduced thalamic volume may reflect atypical brain development, which in turn seems to impact long-term neurodevelopmental outcome. These findings warrant further research, which could lead to tailoring interventions and strategies starting during early postnatal life to promote optimal neurodevelopment.

## Data availability statement

The raw data supporting the conclusions of this article will be made available by the authors, without undue reservation.

## Ethics statement

The studies involving humans were approved by Comité Coordinador de Ética de la Investigación Biomédica de Andalucía. The studies were conducted in accordance with the local legislation and institutional requirements. Written informed consent for participation in this study was provided by the participants' legal guardians/next of kin.

## Author contributions

ET: Conceptualization, Data curation, Formal analysis, Investigation, Methodology, Visualization, Writing – original draft, Writing – review & editing. BJ: Conceptualization, Data curation, Formal analysis, Methodology, Software, Writing – review & editing. NJ-L: Data curation, Writing – review & editing. YM: Data curation, Writing – review & editing. MR: Data curation, Writing – review & editing. ML: Data curation, Writing – review & editing. ER: Data curation, Writing – review & editing. AS: Data curation, Writing – review & editing. SL-L: Conceptualization, Methodology, Supervision, Writing – review & editing. IB-F: Conceptualization, Methodology, Supervision, Writing – review & editing.
